# Predictors of very preterm births (born between 23 and 29 weeks’ gestation) at a tertiary care center in Karachi, Pakistan: additional multivariate analyses on data from primary cohort

**DOI:** 10.1186/s13104-019-4647-8

**Published:** 2019-09-23

**Authors:** Mohammad Yawar Yakoob

**Affiliations:** Indus Hospital Research Center, Indus Health Network, Karachi, Pakistan

**Keywords:** Very preterm infants, Predictors, Gestational age, Survival, Incidences, Pakistan

## Abstract

**Objective:**

Previously, we have published univariate analyses on a cohort of all singleton, very preterm infants (N = 101) born between 23 and 29 weeks of gestation during January 01, 1998 to June 30, 2003 at The Aga Khan University Hospital in Karachi, Pakistan. Our main objective was to extend these analyses to multivariate logistic regression models and report odds ratios (ORs) for univariate and multivariate analyses. All variables in univariate were included in multivariate models.

**Results:**

The survival incidences were 0% at 23, 16.7% at 24, 40.0% at 25, 30.0% at 26, 33.3% at 27, 68.8% at 28 and 83.9% at 29 weeks of gestation. In univariate analyses, gestational age, birth-weight and mode of delivery (cesarean-section had higher survival compared to vaginal) were statistically significant predictors of survival (P ≤ 0.001 each). Other variables that also included antenatal steroids did not achieve significance. However, in complete-case multivariate analyses, only gestational age (per week) was associated with survival (OR = 2.5, 95% CI 1.1–5.5, P = 0.03); birth-weight (per 100 g) and C-section were not associated-1.2, 0.88–1.6, P = 0.26 and 2.4, 0.48–12.2, P = 0.28. Antenatal steroid use, maternal age, year of birth, parity, history of preterm delivery, hemoglobin levels, complications and time of birth remained not associated.

## Introduction

Very preterm births are considered a serious, life-threatening medical emergency associated with innumerable complications and difficult decision making to prolong survival [1]. Despite advancements in care over the past several decades and improved survival, mortality and morbidity remain high, particularly at the lowest gestational ages [2]. These births are associated with short- and long-term complications. In the first few weeks, the complications may include breathing, heart, brain, gastrointestinal, temperature control, blood, metabolism and immune system problems. Long-term complications are related to neurodevelopmental disorders such as cerebral palsy, impaired learning, vision and hearing problems, etc. [3]. Very preterm births also incur high costs as they require specialized care in intensive care nurseries and if survive beyond early months, then costs associated with health and education during the early years [4].

Appropriate statistics are necessary for the decision making process of the obstetrician and to counsel parents and guardians regarding the probability of survival given a particular gestational age. Identifying predictors of survival will assist in dividing infants into high risk categories. Data on complications will enable obstetrician to know what to anticipate after delivery and prepare for interventions to ameliorate or treat these complications. There is paucity of data from Pakistan, including our hospital, on survival and predictors dictating survival of very preterm births despite its large burden in developing countries. Previously, we have published analyses on a cohort of all singleton very preterm infants born between 23 and 29 weeks of gestation during January 01, 1998 to June 30, 2003 at The Aga Khan University Hospital in Karachi, Pakistan [5]. We conducted univariate analyses without reporting odds ratios (ORs) and also only reported prevalences of survival according to gestational age and birth-weight. Our main objective was to extend these analyses to conduct multivariate logistic regression models and also report ORs for both univariate and multivariate analyses.

## Main text

### Methods

The study base comprised of singleton very preterm infants born during the defined period above at this tertiary care center. The antenatal, perinatal and postnatal data were collected through medical records review of these infants and their mothers. The hospital course and complications of both infants and mothers were studied. Gestational age was determined from the mother’s first day of last menstrual period and early first trimester ultrasound scan, where available.

We conducted both univariate and multivariate logistic regression analyses for different explanatory (independent) variables with survival as outcome. Univariate analyses were confirmed by Chi-square or Fisher’s exact test. All potential predictor variables were included in multivariate models. P < 0.05 was considered statistically significant and P = 0.05–0.10 was considered associated with a trend only. STATA 14.0 software was used for statistical analyses.

### Results

101 very preterm infants were born during the defined study period. The mean maternal age was 28.1 ± 5.1 years and mean hemoglobin (Hb) of 11.0 ± 1.4 g/dL (n = 78/101) at the time of delivery; 41.6% of mothers had Hb ≤ 11 g/dL i.e. were anemic. 26 mothers had chronic hypertension, pregnancy-induced hypertension/pre-eclampsia or eclampsia and 10 mothers had pre-existing or gestational diabetes. The cause of preterm delivery was identified in 97 mothers and was preterm premature rupture of membranes (40/97, 41.2%), followed by pre-eclampsia/eclampsia 17/97 (17.5%), antepartum hemorrhage 12/97 (12.4%), fetal distress/oligohydramnios/intra-uterine growth restriction 6/97 (6.2%), preterm labor 5/97 (5.2%), cervical incompetence/cerclage 4/97 (4.1%), hepatic failure/HELLP syndrome 3/97 (3.1%), maternal cancer 3/97 (3.1%), per-vaginal bleed 2/97 (2.1%), hydrocephalus/myelomeningocele 2/97 (2.1%), septate/fibroid uterus 2/97 (2.1%) and cord prolapse 1/97 (1.0%).

The mean birth weight of these infants was 1042.1 ± 304.8 g (range = 400–1750). 53 (52.5%) were males. 54/101 (53.5%) were born via cesarean section and 47/101 (46.5%) had vaginal delivery. The presentation at delivery was cephalic in more than half of infants (60/95, 63.2%). 76/101 (75.3%) of infants required intubation. The median Apgar score was 5 at 1 min and 7 at 5 min. Other detailed characteristics of these infants have been published elsewhere [5]. The survival incidences were 0% at 23, 16.7% at 24, 40.0% at 25, 30.0% at 26, 33.3% at 27, 68.8% at 28 and 83.9% at 29 weeks of gestation. The corresponding survival proportions were 0%, 33.3%, 55.8%, 80.6% and 100% for birth weight categories of < 400, 400–800, > 800–1200, > 1200–1600 and > 1600–2000 g. Overall, 59/101 (58.4%) of infants survived to hospital discharge.

In univariate analyses, gestational age (per 1 week increase, OR = 2.0, 95% confidence interval (CI) 1.4–2.9); birth weight (per 100 g increase, OR = 1.4, 1.2–1.6) and mode of delivery (cesarean section had higher survival compared to vaginal, OR = 4.2, 1.8–9.8) were statistically significant predictors of survival (P ≤ 0.001 each) (Table 1). Other variables that also included antenatal steroids (Reference: 0 mg; 12 mg OR = 3.2, 0.75–13.2, P = 0.12; 24 mg OR = 2.0, 0.75–5.5, P = 0.16; > 24 mg OR = 3.9, 0.95–15.7, P = 0.06) did not achieve significance. In complete case multivariate analyses after excluding n = 23 missing values for Hb at delivery, only gestational age (in per 1 week) was associated with survival with statistical significance, OR = 2.5 (95% CI 1.1–5.5), P = 0.03. Birthweight (per 100 g increase) and abdominal delivery were not associated-OR = 1.2 (0.88–1.6), P = 0.26 and 2.4 (0.48–12.2), P = 0.28, respectively (Table 1). Antenatal steroid use, maternal age, year of birth, parity, history of preterm delivery, Hb at delivery, time of birth and any of maternal complications (presence of hypertension or diabetes during pregnancy) remained not associated. After removing Hb as a variable in the multivariate model and retaining all N = 101 observations, gestational age remained associated (OR = 1.9, 1.1–3.4, P = 0.03) and maternal complications was associated with a trend towards reduced odds of survival (OR = 0.30, 0.08–1.1, P = 0.07).

**Table 1:** Univariate and multivariate predictors of survival of very preterm infants in our study (N = 101)

Variables	Univariate OR (95% CI)	P-value	Multivariate OR (95% CI) (N = 78)	P-value	Multivariate OR (95% CI) after removing Hb variable (N = 101)	P-value
Gestational age (per 1 week increase)	2.0 (1.4–2.9)	< 0.001	2.5 (1.1–5.5)	0.03	1.9 (1.1–3.4)	0.03
Birth weight in grams (per 100 g increase)	1.4 (1.2–1.6)	< 0.001	1.2 (0.88–1.6)	0.26	1.2 (0.93–1.5)	0.19
C-section vs. vaginal delivery	4.2 (1.8–9.8)	0.001	2.4 (0.48–12.2)	0.28	2.1 (0.67–6.8)	0.20
Steroid use (mg)						
0	Reference		Reference		Reference	
12	3.2 (0.75–13.2)	0.12	7.2 (0.59–86.9)	0.12	3.4 (0.49–23.9)	0.22
24	2.0 (0.75–5.5)	0.16	1.3 (0.25–7.2)	0.74	1.3 (0.31–5.6)	0.71
> 24	3.9 (0.95–15.7)	0.06	0.60 (0.04–7.9)	0.70	2.9 (0.43–19.0)	0.28
Maternal age in years (per unit increase)	1.1 (0.99–1.2)	0.11	1.1 (0.97–1.3)	0.12	1.1 (0.96–1.2)	0.19
Year of birth (per year increase)	1.2 (0.92–1.6)	0.18	0.95 (0.56–1.6)	0.86	1.1 (0.71–1.6)	0.73
Maternal parity						
Primiparous	Reference		Reference		Reference	
2–4	0.88 (0.38–2.0)	0.77	1.3 (0.23–7.2)	0.77	0.59 (0.15–2.4)	0.45
5–9	0.82 (0.19–3.5)	0.79	0.80 (0.03–18.5)	0.89	0.43 (0.05–3.6)	0.44
History of preterm delivery						
0	Reference		Reference		Reference	
≥ 1	0.77 (0.30–1.96)	0.58	0.88 (0.13–6.0)	0.90	1.3 (0.30–5.4)	0.73
Hb at delivery (per unit increase) [missing n = 23]	1.1 (0.77–1.5)	0.64	1.1 (0.60–1.9)	0.80	–	–
Time of birth						
0000 to < 0800	Reference		Reference		Reference	
0800 to < 1700	1.9 (0.69–5.1)	0.22	7.4 (1.0–54.7)	0.05	2.5 (0.56–11.3)	0.23
1700 to 2400	1.5 (0.51–4.7)	0.44	3.4 (0.43–27.5)	0.25	3.0 (0.62–14.4)	0.17
Any of maternal complications (hypertension or diabetes)	0.86 (0.37–2.0)	0.71	0.22 (0.04–1.4)	0.12	0.30 (0.08–1.1)	0.07

### Sensitivity analyses

After removing birthweight from the multivariable model, the gestational age variable again became highly statistically significant (OR = 2.4, 1.5–3.9, P < 0.001) as in univariate analysis showing that the effect of gestational age was partially mediated through birthweight. Similarly, removing gestational age from the model also made birthweight and abdominal delivery statistically significant (OR = 1.4, 1.2–1.7, P = 0.001) and (OR = 3.6, 1.2–10.4, P = 0.02) indicating that gestational age may be a confounder.

## Limitations

The medical records are designed for clinical and not research purposes so there may have been misclassification in reporting data, particularly on antenatal steroids. It may have also been possible that the mother may have been given steroids but not written in the medical records by the intern/resident/obstetrician. We also did not collect data on the proportion of pregnancies where gestational age was confirmed by early ultrasound scan but records according to gestational age were obtained through ICD code and these were the most valid estimates available. The number of survival events relative to the number of predictors were few so the study could have been underpowered but the multivariate models still managed to converge. The data is from the last decade so the survival incidences may have changed over time due to advancement in pediatric/neonatal intensive care units at this institute.

In conclusion, gestational age, birth weight and C-section (vs. vaginal delivery) were statistically significant predictors of infant survival in univariate analyses. However, gestational age was the only variable that remained associated with survival in multivariate analyses (the effect of which was partially mediated through birth-weight); while birth-weight and C-section became non-significant.

## Data Availability

The data can be shared on request to the author at myawaryakoob@gmail.com.

## Abbreviations

OR: odds ratio
Hb: hemoglobin
HELLP: hemoloysis, elevated liver enzymes and low platelet count syndrome
C-section: cesarean section
ICD: International Classification of Diseases
