# Correction to: Predictors of survival of very preterm births (born between 23 and 29 weeks’ gestation) at a tertiary care center in Karachi, Pakistan: additional multivariate analyses on data from primary cohort

**DOI:** 10.1186/s13104-020-05004-6

**Published:** 2020-03-23

**Authors:** Mohammad Yawar Yakoob

**Affiliations:** Indus Hospital Research Center, Indus Health Network, Karachi, Pakistan

## Correction to: BMC Res Notes (2019) 12:620 10.1186/s13104-019-4647-8

For better clarity, the author would like to amend the title of the original article [1].

“Predictors of very preterm births (born between 23 and 29 weeks’ gestation) at a tertiary care center in Karachi, Pakistan: additional multivariate analyses on data from primary cohort”

should be read as:

“Predictors of survival of very preterm births (born between 23 and 29 weeks’ gestation) at a tertiary care center in Karachi, Pakistan: additional multivariate analyses on data from primary cohort”.

The corrected title has been included in this Correction article.

## References

[1] Yakoob MY (2019). Predictors of very preterm births (born between 23 and 29 weeks’ gestation) at a tertiary care center in Karachi, Pakistan: additional multivariate analyses on data from primary cohort. BMC Res Notes.

